# Reduced IgG anti-small nuclear ribonucleoprotein autoantibody production in systemic lupus erythematosus patients with positive IgM anti-cytomegalovirus antibodies

**DOI:** 10.1186/ar2621

**Published:** 2009-02-20

**Authors:** Claudia Azucena Palafox Sánchez, Minoru Satoh, Edward KL Chan, Wendy C Carcamo, José Francisco Muñoz Valle, Gerardo Orozco Barocio, Edith Oregon Romero, Rosa Elena Navarro Hernández, Mario Salazar Páramo, Antonio Cabral Castañeda, Mónica Vázquez del Mercado

**Affiliations:** 1 Departamento de Biología Molecular y Genómica, Instituto de Investigación en Reumatología y del Sistema Músculo Esquelético (IIRSME), Centro Universitario de Ciencias de la Salud, Universidad de Guadalajara, Sierra Mojada 950, Guadalajara, Jalisco, CP 44340, México; 2 Division of Rheumatology and Clinical Immunology, Department of Medicine, University of Florida, P.O. Box 100221, Gainesville, FL 32610-0221, USA; 3 Department of Pathology, Immunology, and Laboratory Medicine, University of Florida, P.O. Box 100221, Gainesville, FL 32610-0221, USA; 4 Department of Oral Biology, University of Florida, 1600 SW Archer Road, Gainesville, FL 32610-0424, USA; 5 Departamento de Inmunología y Reumatología del Hospital General de Occidente, Secretaría de Salud Jalisco, Av. Zoquipan 1050, CP 45100, Zapopan, Jalisco, México; 6 División de Investigación, Hospital de Especialidades, CMNO, IMSS, and Deparamento de Fisiología, CUCS, Universidad de Guadalajara, Sierra Mojada 950, CP 4430, Guadalajara, Jalisco, México; 7 Departamento de Reumatología, Instituto Nacional en Ciencias Médicas y de la Nutrición Salvador Zubirán, Vasco de Quiroga 15, Tlalpan C.P. 14000, Mexico, DF, México; 8 División de Medicina Interna, Departamento de Reumatología, Hospital Civil Juan I Menchaca, Salvador de Quevedo y Zubieta N° 750, CP 44340, Guadalajara, Jalisco, México

## Abstract

**Introduction:**

Systemic lupus erythematosus is characterized by production of autoantibodies to RNA or DNA–protein complexes such as small nuclear ribonucleoproteins (snRNPs). A role of Epstein–Barr virus in the pathogenesis has been suggested. Similar to Epstein–Barr virus, cytomegalovirus (CMV) infects the majority of individuals at a young age and establishes latency with a potential for reactivation. Homology of CMV glycoprotein B (UL55) with the U1snRNP-70 kDa protein (U1–70 k) has been described; however, the role of CMV infection in production of anti-snRNPs is controversial. We investigated the association of CMV serology and autoantibodies in systemic lupus erythematosus.

**Methods:**

Sixty-one Mexican patients with systemic lupus erythematosus were tested for CMV and Epstein–Barr virus serology (viral capsid antigen, IgG, IgM) and autoantibodies by immunoprecipitation and ELISA (IgG and IgM class, U1RNP/Sm, U1–70 k, P peptide, rheumatoid factor, dsDNA, β_2_-glycoprotein I).

**Results:**

IgG anti-CMV and IgM anti-CMV were positive in 95% (58/61) and 33% (20/61), respectively, and two cases were negative for both. Clinical manifestation and autoantibodies in the IgM anti-CMV(+) group (n = 20) versus the IgM anti-CMV(-)IgG (+) (n = 39) group were compared. Most (19/20) of the IgM anti-CMV(+) cases were IgG anti-CMV(+), consistent with reactivation or reinfection. IgM anti-CMV was unrelated to rheumatoid factor or IgM class autoantibodies and none was positive for IgM anti-Epstein–Barr virus–viral capsid antigen, indicating that this is not simply due to false positive results caused by rheumatoid factor or nonspecific binding by certain IgM. The IgM anti-CMV(+) group has significantly lower levels of IgG anti-U1RNP/Sm and IgG anti-U1–70 k (*P *= 0.0004 and *P *= 0.0046, respectively). This finding was also confirmed by immunoprecipitation. Among the IgM anti-CMV(-) subset, anti-Su was associated with anti-U1RNP and anti-Ro (*P *< 0.05). High levels of IgG anti-CMV were associated with production of lupus-related autoantibodies to RNA or DNA–protein complex (*P *= 0.0077).

**Conclusions:**

Our findings suggest a potential role of CMV in regulation of autoantibodies to snRNPs and may provide a unique insight to understand the pathogenesis.

## Introduction

Systemic lupus erythematosus (SLE) is an autoimmune disease of unknown etiology, characterized by production of autoantibodies to cellular constituents – in particular, complexes of dsDNA or RNA and proteins [1]. Various genetic and environmental factors appear to be involved in the development of SLE and the production of autoantibodies. Among the environmental factors, a role of viruses in triggering SLE has been investigated for many years [2,3]. However, traditional approaches to identify unique viruses among SLE patients did not produce consistent results, however, and recent evidence suggests that common viruses such as Epstein–Barr virus (EBV), cytomegalovirus (CMV), and parvovirus B19, to which many individuals are exposed during life, may play a role in the pathogenesis of SLE [2,3]. Increased prevalence of EBV infection among SLE patients [4], homology of EBV nuclear antigen (EBNA) 1 antigen and small nuclear ribonucleoproteins (snRNPs) [5], the pattern of epitope spreading consistent with molecular mimicry mechanism of induction of autoantibodies [6], and supporting evidence from animal models have all been described [5,7,8]. Similar to EBV, CMV infects the majority of individuals at a young age and establishes lifelong latency with possible reactivation at various times caused by a variety of triggers such as acute inflammation [9,10]. The reported prevalence of CMV infection based on detection of anti-CMV antibodies or CMV-DNA by PCR analysis of whole blood samples in SLE patients is from 60% to 100% similar to the control population in most studies [11,12]. A new infection or reactivation of CMV can mimic SLE in some cases [12,13].

Previous studies have shown a homology of the U1snRNP-70 kDa protein (U1–70 k) and CMV envelope glycoprotein B (UL55) and induction of anti-U1–70 k antibodies by glycoprotein B in a mouse model [14,15]. Association between autoantibodies to the U1snRNPs and CMV infection in healthy subjects and SLE patients has been reported [11]; however, this was not confirmed in another study [16].

In the present study, we investigated whether the serological status of CMV infection has an association with the production of specific lupus autoantibodies – in particular, antibodies to snRNPs.

## Materials and methods

### Patients

Sixty-one consecutive patients with SLE from the Department of Rheumatology, Hospital General de Occidente, Zapopan, Jalisco, Mexico were studied. All patients fulfilled the 1982 American College of Rheumatology SLE classification criteria [17].

The Mexican Systemic Lupus Erythematosus Disease Activity Index and the Systemic Lupus International Collaborating Clinics/American Collage of Rheumatology Damage Indexes at the beginning of the study were evaluated [18,19]. A complete blood count, including the lymphocyte count and serum rheumatoid factor (RF) (CELL-DYN 3500R; Abbott Diagnostics (Santa Clara, CA, USA), was determined in all subjects. Information on treatment on the day of sampling, including use of immunosuppressive drugs (azathioprine, methotrexate, and cyclophosphamide), chloroquine, and a dose of steroid (prednisone mg/day), was recorded.

The protocol was approved by the Institutional Review Board. The present study meets and is in compliance with all ethical standards in medicine, and written informed consent was obtained from all patients according to the Declaration of Helsinki.

### Viral serology

IgG and IgM antibodies against CMV were measured using a microparticle enzyme immunoassay kit (Abbott Laboratories, Abbott Park, IL, USA) following the manufacturer's instructions. The specimens with index values ≥ 0.5 units/ml and ≥ 15 units/ml, respectively, for IgM and IgG antibodies to CMV, were considered positive. IgG and IgM antibodies to EBV viral capsid antigen (VCA) were measured by ELISA (Biotech Atlantic Inc., Eatontown, NJ, USA).

### Anti-U1RNP/Sm antigen-capture ELISA

Anti-U1RNP/Sm antigen-capture ELISA was performed essentially as described for other human autoantibody systems [20]. Briefly, microtiter plates (Immobilizer Amino™; Nalgene Nunc [Rochester, NY, USA]) were coated with 3 μg/ml mouse mAb 2.73 (IgG_2a_, anti-U1–70 k) [21] overnight and were blocked with 0.5% BSA NET/NP40 (150 mM NaCl, 2 mM ethylenediamine tetraacetic acid, 50 mM Tris–HCl, pH 7.5, 0.3% Nonidet-P40). The left half of the plate was incubated with K562 cell lysate (50 μl/well, 4 × 10^7^/ml) and the right half was incubated with the blocking buffer. After washing the plate with Tris-buffered saline/Tween20 (20 mM Tris–HCl, pH 7.5, 150 mM NaCl, 0.1% Tween20) three times and 0.5 M NaCl/NET/NP40 (500 mM NaCl, 2 mM ethylenediamine tetraacetic acid, 50 mM Tris–HCl, pH 7.5, 0.3% Nonidet-P40) three times, an identical set of samples and serially diluted standard serum (1:500 to serial 1:5 dilutions) were added to the left half and the right half (control for reactivity against mouse IgG) of the plate. Serum samples were tested at 1:500 and 1:2,500 dilutions, and data from the latter were used for the analysis. Plates were washed with Tris-buffered saline/Tween20, incubated with alkaline phosphatase-conjugated mouse mAbs to human IgG (1:1,000 dilution; Sigma [St. Louis, MO, USA]) and developed. The 405 nm optical density of wells were converted into units based on the standard curve using SoftMax Pro 4.3 software (Molecular Devices, Sunnyvale, CA, USA) and the units of the corresponding right half (without U1RNP/Sm antigens) were subtracted from those of the left half (with antigens) [20].

### ELISA for antibodies to P peptide, dsDNA, mouse IgG (rheumatoid factor), U1–70 k, and β_2_-glycoprotein I

Microtiter plates (Immobilizer Amino™; Nunc) were incubated with 1 to 3 μg/ml appropriate antigen, and ELISA was performed as described previously using 1:500 (for all IgG class antibodies), 1:2,500 (IgG anti-U1–70 k ELISA) or 1:100 (for all IgM class antibodies) diluted sera. Optical densities were converted into units as described using appropriate standard [20]. P peptide was the COOH-terminal 22 amino acids of human P0 protein [22]. Mouse IgG was a mixture of mouse IgG_1 _and IgG_2a _myeloma proteins (Southern Biotechnology, Birmingham, AL, USA). dsDNA was purified using S1 nuclease and the ELISA was performed as described previously [20]. β_2_-glycoprotein I was a gift from Dr Jyunichi Kaburaki (Tokyo Electric Company Hospital, Tokyo, Japan).

U1–70 k recombinant protein was a 184 amino acid fragment spanning amino acids 240 to 423, a major human B-cell epitope that contains the arginine/serine-rich region [23]. The full-length cDNA for U1–70 k protein was kindly provided by Dr Ger JM Pruijn (Department of Biochemistry, University of Nijmegen, The Netherlands). The cDNA fragment was cloned by PCR amplification and subcloned into pDONR transition vector (Invitrogen, Carlsbad, CA, USA) and then into pDEST17 vector. The recombinant protein was expressed in *Escherichia coli *and purified via nickel affinity column.

### Screening of autoantibodies in human sera by immunoprecipitation

Immunoprecipitation using ^35^S-methionine-labeled K562 cell extract was performed using 8 μl sera as described elsewhere [21]. Specificities were determined using previously described reference sera. Positive anti-U1RNP was defined based on the presence of the set of U1RNP proteins (A, B'/B, C, D1/D2/D3, E/F, and G). Since autoantibodies to U5RNP without anti-Sm are very rare [24], immunoprecipitation of the characteristic U5RNP 200 kDa proteins was used to define anti-Sm (which immunoprecipitates U2, U4–U6, and U5 in addition to U1RNP) [21].

### Statistical analysis

All statistical analysis was performed using Prism 5.0 for Macintosh (GraphPad Software, Inc., San Diego, CA, USA). Fisher's exact test and the Mann–Whitney test were used to analyze the prevalence and the levels, respectively, of autoantibodies and other data.

## Results

### Cytomegalovirus serology

The prevalence of positive IgG anti-CMV antibodies and positive IgM anti-CMV antibodies in the SLE population was 95% (58/61) and 33% (20/61), respectively. Two subjects, negative for both IgG and IgM antibodies, were excluded from the following analysis. The rest of the SLE patients were divided into two groups; 20 subjects who were anti-CMV IgM(+) [19 IgG(+) and one IgG(-)], and 39 subjects who were anti-CMV IgM(-)IgG(+) (Table 1).

**Table 1:** Demographic and clinical characteristics in systemic lupus erythematosus patients with IgM anti-CMV(+) versus IgM anti-CMV(-)

	All patients	IgM anti-CMV(+) patients	IgM anti-CMV(-) IgG anti-CMV(+) patients
*n*	59	20^a^	39
Age (years)	36.7 ± 14.1 (13 to 72)	34.3 ± 15.1 (14 to 72)	37.9 ± 13.6 (13 to 64)
Sex (female/male)	56/3	18/2	38/1
Disease status			
Disease duration (years)	7.4 ± 6.4 (0.1 to 36)	9.4 ± 8.0 (2 to 36)	6.5 ± 5.3 (0.1 to 23)
Age at diagnosis (years)	29.2 ± 12.1 (7 to 57)	24.9 ± 10.9* (7 to 57)	31.5 ± 12.3* (12 to 57)
Clinical assessment			
Mexican SLEDAI	2.88 ± 2.97 (0.0 to 12)	3.30 ± 3.26 (0.0 to 10)	2.67 ± 2.83 (0.0 to 12)
SLICC	0.76 ± 1.75 (0.0 to 12)	1.11 ± 2.75 (0.0 to 12)	0.59 ± 0.97 (0.0 to 3)
Laboratory data			
Rheumatoid factor (IU/ml)	18.2 ± 20.9 (2 to 108)	21.3 ± 29.0 (2 to 108)	16.7 ± 15.5 (3 to 60)
Soluble IL-10 (pg/ml)	27.9 ± 54.8 (9.31 to 422)	39.4 ± 90.5 (11.7 to 422)	22.0 ± 19.4 (9.3 to 126.7)
IgG CMV (U)	233.7 ± 46.3 (3.5 to 250)	233.6 ± 55.3 (3.5 to 250)	233.8 ± 41.8 (70 to 250)
White blood cells (/μl)	5,782 ± 2,008 (2,870 to 12,100)	5,413 ± 1,713 (2,900 to 8,700)	5,962 ± 2,135 (2,870 to 12,100)
Lymphocyte (/μl)	1493 ± 688 (154 to 3,400)	1450 ± 676 (154 to 2,890)	1515 ± 702 (269 to 3,400)
Platelet (× 10^3^/μl)	273.8 ± 63.9 (137 to 445)	262.8 ± 81.7 (137 to 445)	279.2 ± 53.7 (167 to 385)
Treatment			
Azathioprine (%)	59	47	64
Methotrexate (%)	21	16	23
Cyclophosphamide (%)	5	5	5
Immunosuppressive therapy^b ^(%)	79	63	87
Chloroquine (%)	52	53	51
On steroid (%)	57	68	51
Steroid dose (prednisone mg/day)	4.7 ± 6.1	5.9 ± 5.9	4.1 ± 6.2
Prednisone ≥ 10 mg/day (%)	17	26	13

Data presented as the mean ± standard deviation (minimum to maximum). All values were compared between the IgM anti-CMV(+) group and the IgM anti-CMV(-)IgG(+) group using the Mann–Whitney test (values) or Fisher's exact test (%). *P *values were not significant (*P *> 0.05) except for age at diagnosis (*P = 0.048). CMV, cytomegalovirus; SLEDAI, Systemic Lupus Erythematosus Disease Activity Index; SLICC, Systemic Lupus International Collaborating Clinics. ^a^Information for treatment was available in 19 patients in this group. ^b^Azathioprine, methotrexate, or cyclophosphamide.

### Demographic and clinical characteristics

The demographic and clinical characteristics of the subjects comparing the IgM anti-CMV(+) group versus the IgM anti-CMV(-)IgG(+) group are summarized in Table 1. Of these 59 patients, 56 were female and three were male with mean age of 36.7 ± 14.1 years. Age at onset was younger in the IgM anti-CMV(+) group (*P *< 0.05 by Mann–Whitney test) but otherwise no significant differences were observed between the groups. The percentage of patients on immunosuppressive drugs (azathioprine, methotrexate, and cyclophosphamide), on chloroquine, or on steroids, and a dose of steroids (prednisone mg/day) also was not significantly different between groups.

### IgM anti-CMV antibodies are not due to rheumatoid factor or nonspecific reactivity of certain IgM antibodies

IgM anti-CMV can become positive in some cases of reactivation or reinfection of CMV [9,10]. The prevalence of IgM anti-CMV among SLE patients in the present study was much higher (33%) than in the general population, similar to some previous studies that also reported high prevalence of IgM anti-CMV among SLE patients [25-27]. False positive results for IgM anti-CMV due to RF or nonspecific binding of certain IgM antibodies, however, have also been reported [25].

**Figure 1:**
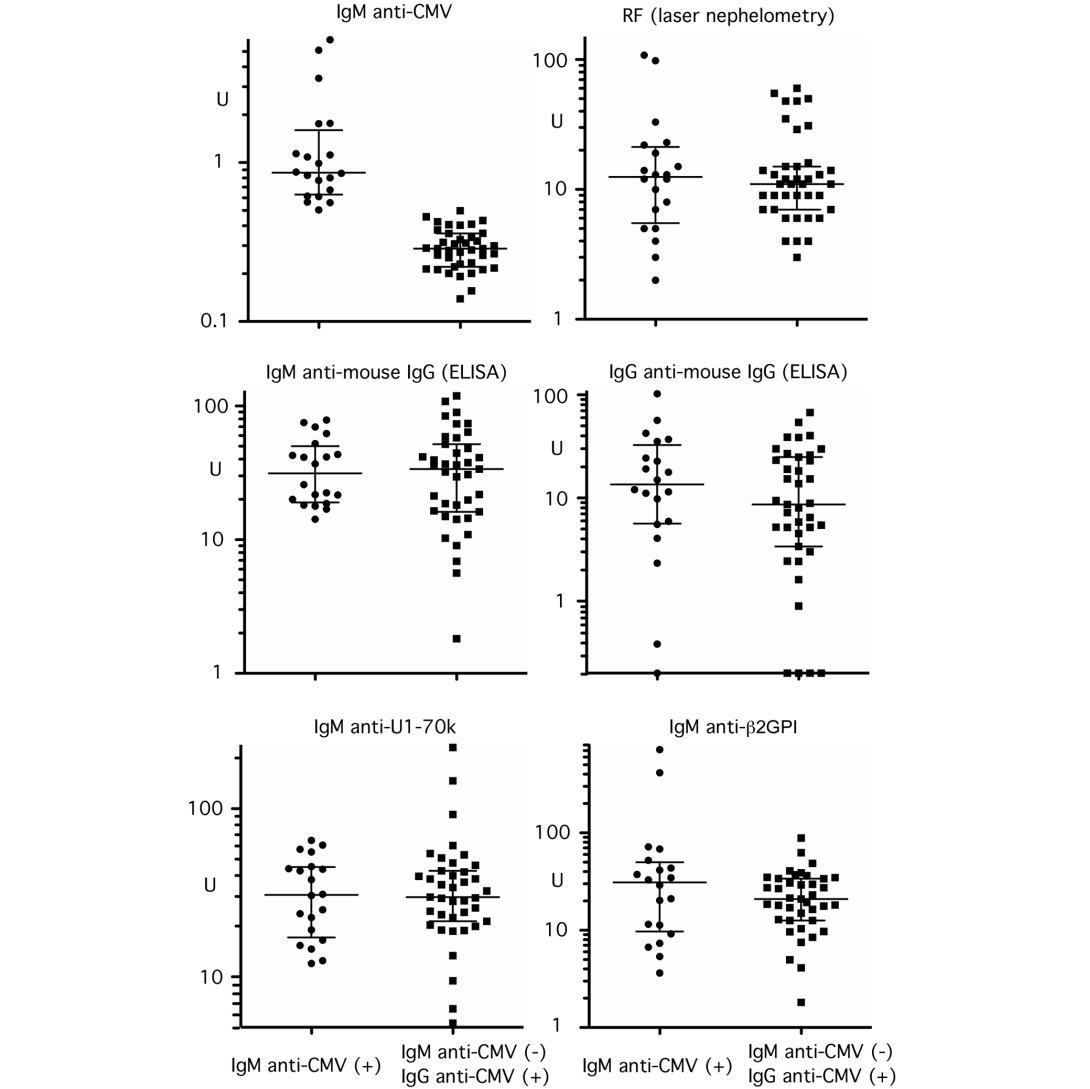
Serum rheumatoid factor and other IgM class antibodies in IgM anti-CMV(+) versus anti-CMV(-) patients. IgM anti-cytomegalovirus (CMV) (microparticle enzyme immunoassay), rheumatoid factor (RF) (laser nephelometry), IgM or IgG antibodies to mouse IgG, IgM anti-U1snRNP-70 kDa protein (anti-U1–70 k) and anti-β_2_-glycoprotein I (anti-β2GPI) (ELISA) were performed as described in Materials and methods. Serum dilution for ELISA was 1:100 for IgM class and 1:500 for IgG anti-mouse IgG.

The levels of RF and other control IgM autoantibodies were therefore compared between IgM anti-CMV(+) patients versus IgM anti-CMV(-) patients (Figure 1) to examine whether RF or nonspecific reaction of IgM in certain patients can explain IgM anti-CMV in our cohort. Levels of RF by laser nephelometry and of IgM and IgG RF by ELISA were not higher in the IgM anti-CMV(+) group versus the IgM anti-CMV(-) group, indicating that the positive IgM anti-CMV is not simply due to RF in this group. Furthermore, levels of IgM antibodies to U1–70 k or to β_2_-glycoprotein I (Figure 1), dsDNA, P peptide, U1RNP/Sm, and chromatin (data not shown) were not higher in the IgM anti-CMV(+) group. In addition, all 20 IgM anti-CMV(+) patients were IgM anti-EBV viral capsid antigen-negative, indicating that high levels of IgM anti-CMV in this group are not due to nonspecific high reactivity of IgM antibodies in this group of patients. The IgM anti-CMV(+) group of patients was therefore considered to mainly reflect the reactivation or reinfection of CMV, and possibly some of the patients may be at a late stage of primary infection [one subject was anti-CMV IgM(+)IgG(-)].

### Prevalence and levels of autoantibodies in anti-CMV IgM(+) versus anti-CMV IgM(-)IgG(+) groups

The prevalence and levels of autoantibodies in the IgM anti-CMV(+) group versus the IgM anti-CMV(-)IgG anti-CMV(+) group were compared by immunoprecipitation and ELISA to evaluate the association of the CMV serology and autoantibodies. The prevalence of autoantibodies by immunoprecipitation is summarized in Table 2. In general, the prevalence of autoantibodies was similar except that anti-Sm was found only in the IgM anti-CMV(-) group (15%, 6/39) and none in the IgM anti-CMV(+) group (0/20, not significant). The prevalence of anti-Sm among the anti-U1snRNP-positive subset, however, was significantly lower in the IgM anti-CMV(+) group versus the IgM anti-CMV(-)IgG anti-CMV(+) group (0% vs. 50%, *P *= 0.04 by Fisher's exact test). Furthermore, the prevalence of high levels (>25 units) of antibodies to U1RNP/Sm by ELISA was significantly lower in the anti-CMV IgM(+) versus anti-CMV IgM(-)IgG(+) group (0% vs. 28%, *P *= 0.0106 by Fisher's exact test).

**Table 2:** Frequency of autoantibodies in IgM anti-cytomegalovirus (CMV)(+) patients versus IgM anti-CMV(-) patients

	Anti-CMV IgM(+) patients	Anti-CMV IgM(-)IgG(+) patients
*n*	20	39
U1RNP	37% (7/20)	31% (12/39)
Sm	0% (0/20)	15% (6/39)
Sm/U1RNP	0% (0/7)	50% (6/12)*
Ribosomal P	5% (1/20)	10% (4/39)
RNA helicase A	15% (3/20)	26% (10/39)
Ro	35% (7/20)	38% (15/39)
La	15% (3/20)	8% (3/39)
Ku	10% (2/20)	5% (2/39)
Su	30% (6/20)	23% (9/39)
RNA polymerase II	10% (2/20)	8% (3/39)
U1RNP/Sm ELISA > 25 units	0% (0/20)	28% (11/39)^†^

Sm/U1RNP, prevalence of anti-Sm among anti-U1RNP-positive patients. *P = 0.04. ^†^*P *= 0.0106.

**Figure 2:**
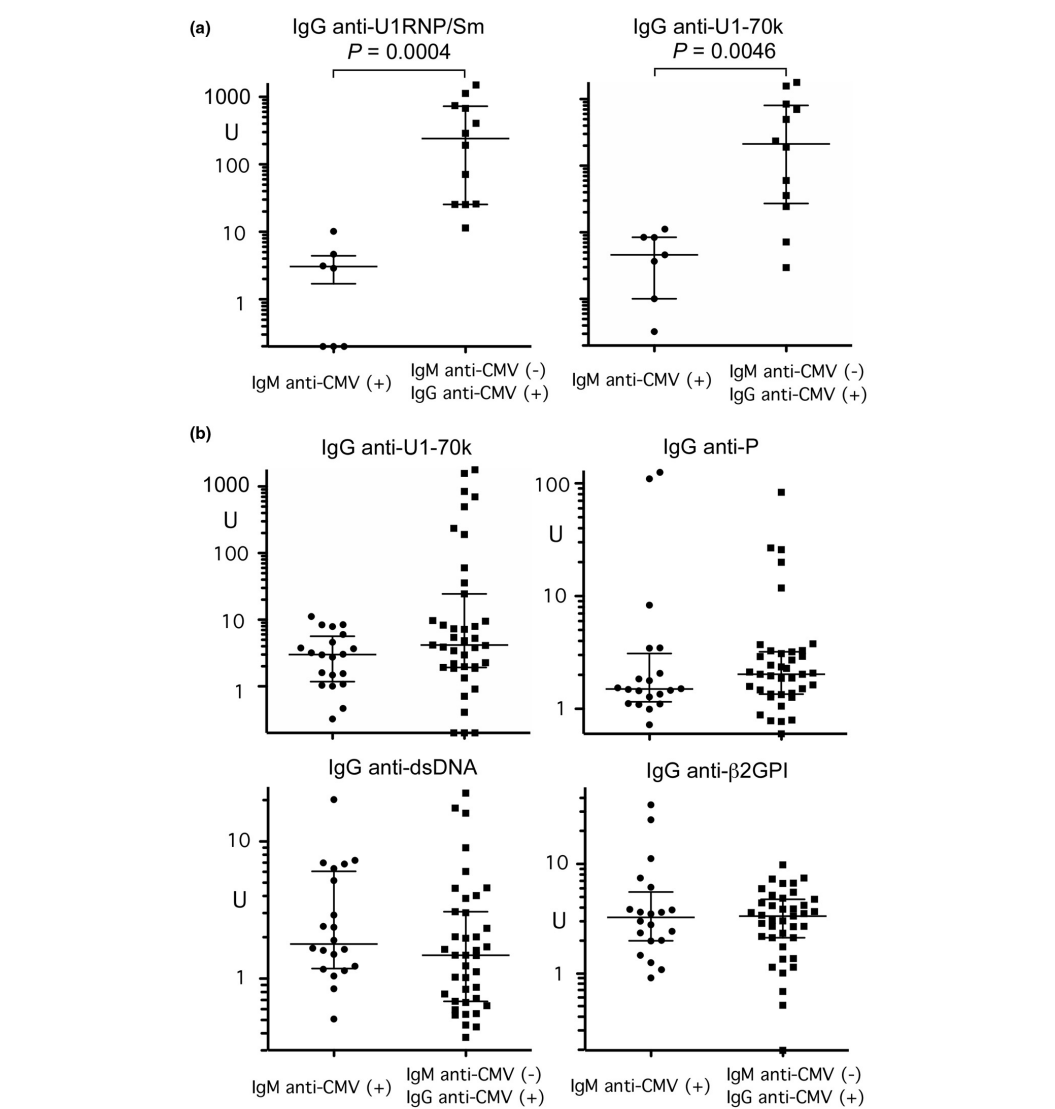
IgG autoantibodies by ELISA in IgM anti-CMV(+) versus IgM anti-CMV(-) patients. **(a) **IgG anti-U1RNP/Sm and IgG anti-U1snRNP-70 kDa protein (anti-U1–70 k) by ELISA. Anti-small nuclear ribonucleoprotein (Anti-snRNP) immunoprecipitation-positive sera (1:2,500 dilution, n = 7 for the IgM anti-cytomegalovirus (CMV)(+) group and n = 12 for the anti-CMV IgM(-)IgG(+) group) were tested for anti-U1RNP/Sm and U1–70 k antibodies by ELISA. **(b) **IgG anti-U1–70 k, P peptide, dsDNA, and anti-β_2_-glycoprotein I (anti-β2GPI) by ELISA. ELISAs were performed as described in Materials and methods using 1:500 diluted sera. For anti-U1RNP/Sm and U1–70 k, data from 1:2,500 diluted sera are shown.

To confirm the difference in levels of autoantibodies to snRNPs, anti-snRNP immunoprecipitation-positive samples (IgM anti-CMV(+) group, n = 7; IgM anti-CMV(-)IgG anti-CMV(+) group, n = 12) were tested by anti-U1RNP/Sm antigen-capture ELISA and anti-U1–70 k ELISA (Figure 2a). The anti-CMV IgM(+) group has significantly lower levels of IgG anti-U1RNP/Sm (*P *= 0.0004) and of IgG anti-U1–70 k (*P *= 0.0046) versus the IgM anti-CMV(-)IgG(+) group. Levels of IgM anti-U1RNP/Sm (data not shown) or IgM anti-U1–70 k (Figure 1, bottom left) were not significantly different between groups, indicating that this is a phenomenon specific for IgG class antibodies. Furthermore, this is specific for IgG anti-U1RNP/Sm and U1–70 k and is not a general phenomenon of IgG-class autoantibodies, since the levels of IgG anti-P peptide, dsDNA, β_2_-glycoprotein I (Figure 2b), or IgG RF were similar between groups (Figure 1).

**Figure 3:**
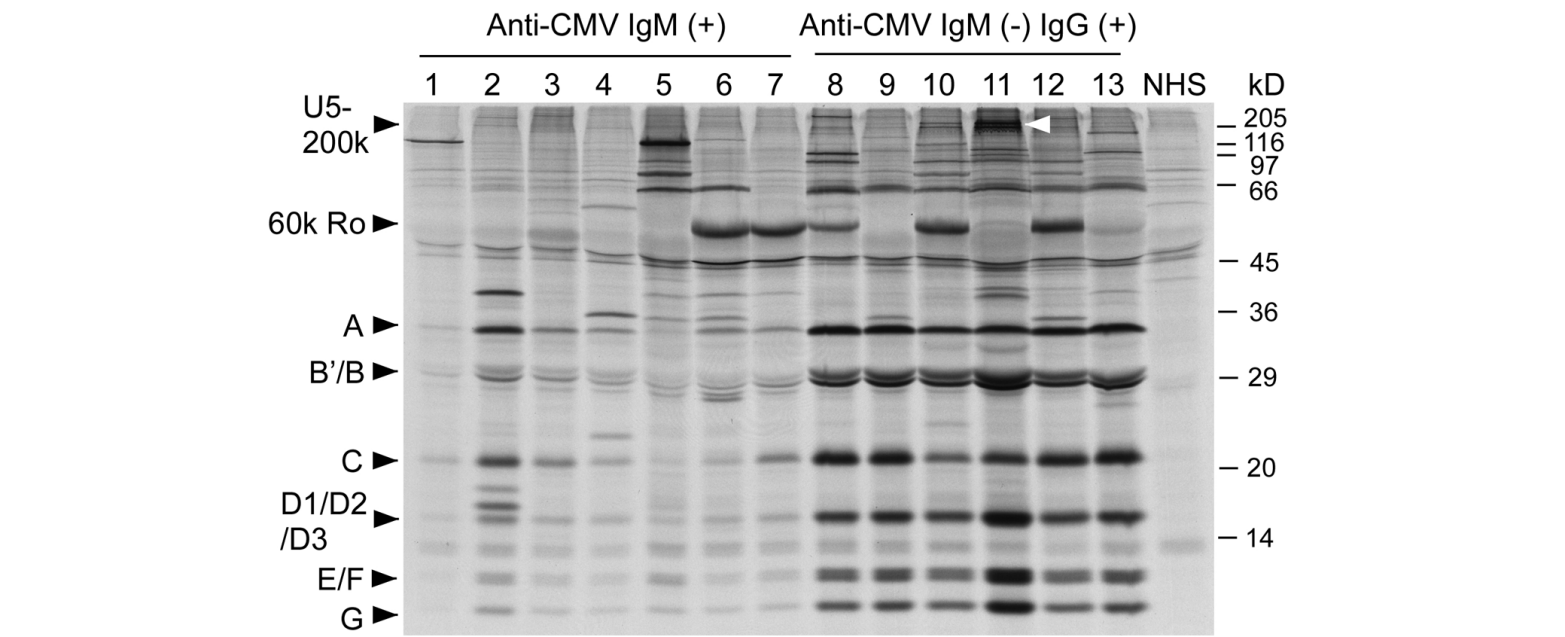
Autoantibodies to small nuclear ribonucleoproteins by immunoprecipitation. Immunoprecipitation by anti-small nuclear ribonucleoprotein immunoprecipitation-positive sera (lanes 1 to 7, all seven sera from the anti-cytomegalovirus (CMV) IgM(+) group; lanes 8 to 13, six randomly selected sera from the 12 in the anti-CMV IgM(-)IgG(+) group) and a normal human serum (NHS) are shown. Positions of UsnRNP components A, B'/B, C, D1/D2/D3, E/F, G, U5–200 k doublet, and 60 k Ro, and molecular weights (kDa) are indicated. White arrowheads, U5–200 k.

Levels of anti-snRNPs were also compared by immunoprecipitation (Figure 3). Although all components of U1snRNPs (A, B'/B, C, D1/D2/D3, E/F, and G) were seen by all seven anti-snRNP-positive sera in the IgM anti-CMV(+) group, signals were much weaker than those by sera from the anti-CMV IgM(-)IgG (+) group, consistent with the ELISA results (Figure 2a). Signals of other autoantibodies by immunoprecipitation – including anti-Ro, ribosomal P, RNA helicase A, and Su – were not clearly different in the IgM anti-CMV(+) group versus the IgM anti-CMV(-)IgG(+) group, consistent with the ELISA data.

All these data indicate that the IgM anti-CMV(+) group has lower levels of IgG autoantibodies specifically for anti-U1RNP/Sm and U1–70 k; however, no difference was observed in IgM-class autoantibodies for these specificities or other autoantibodies in the IgG or IgM class.

### Relationship of different autoantibodies in the anti-CMV IgM(+) and ant-CMV IgM(-)IgG(+) groups

Although the prevalence of various autoantibodies in anti-CMV IgM(+) SLE versus anti-CMV IgM(-)IgG(+) SLE was similar (Table 2), it is possible that the pattern of coexistence of autoantibodies is different. The prevalence of various autoantibodies in anti-U1RNP, Ro, or Su positive versus negative subsets and the presence of IgM anti-CMV antibodies were therefore analyzed. Anti-Su antibodies appeared to frequently coexist with anti-U1RNP or Ro in the anti-CMV IgM(-)IgG(+) group (*P *= 0.0141 and *P *= 0.0631, respectively) but not in the anti-CMV IgM(+) group (Table 3). When similar analysis was performed for anti-U1RNP(+) versus anti-U1RNP(-), anti-Su was found in 50% (6/12) of the former versus 11% (3/27) of the latter (*P *= 0.0141) in the anti-CMV IgM(-)IgG(+) subset. Similarly, anti-Su appeared to be more frequent for anti-Ro(+) than anti-Ro(-), although it did not reach statistical significance (40% vs. 13%, *P *= 0.0631), in the anti-CMV IgM(-)IgG(+) group. Anti-Su and anti-U1RNP or anti-Ro may therefore coexist frequently only in the anti-CMV IgM(-)IgG(+) group of patients.

**Table 3:** Association of anti-Su and other autoantibodies

	Anti-CMV IgM(+) patients		Anti-CMV IgM(-)IgG(+) patients	
		
	Su-positive	Su-negative	Su-positive	Su-negative
*n*	6	14	9	30
U1RNP	17% (1/6)	43% (6/14)	67% (6/9)*	20% (6/30)*
Sm	0% (0/6)	0% (0/14)	33% (3/9)	10% (3/30)
Ribosomal P	17% (1/6)	7% (1/14)	0% (0/9)	13% (4/30)
RNA helicase A	17% (1/6)	14% (2/14)	33% (3/9)	23% (7/30)
Ro	33% (2/6)	36% (5/14)	67% (6/9)^†^	30% (9/30)^2^
La	0% (0/6)	21% (3/14)	11% (1/9)	7% (2/30)

CMV, cytomegalovirus. *P = 0.0141, ^†^*P *= 0.0631.

### Levels of IgG anti-CMV antibodies and presence of autoantibodies

Seventy-seven percent (47/61) of cases had very high levels (>250 units) of IgG anti-CMV; 89% (54/61) of cases had IgG anti-CMV > 200 units, while only seven cases had IgG anti-CMV < 200 units. The prevalence of autoantibodies by immunoprecipitation was compared between these two groups (Table 4). The latter group (IgG anti-CMV < 200 units) had only one each of anti-Ro and anti-Su, and none in this group had anti-U1RNP (0% vs. 35%, *P *= 0.0875), Sm, ribosomal P, RNA helicase A, La, and Ku. Only 28% in this IgG anti-CMV < 200 units group had any of the above listed autoantibodies versus 81% (44/54) in the IgG anti-CMV > 200 units group (*P *= 0.0077 by Fisher's exact test), indicating that the production of IgG lupus-related autoantibodies to RNA or dsDNA–protein complexes is generally more frequent in patients with high levels of IgG anti-CMV.

**Table 4:** Frequency of autoantibodies in low IgG anti-cytomegalovirus (CMV) patients versus high IgG anti-CMV patients

	Low IgG anti-CMV (<200 units)	High IgG anti-CMV (>200 units)
*n*	7	54
U1RNP	0% (0/7)	35% (19/54)*
Sm	0% (0/7)	11% (6/54)
Ribosomal P	0% (0/7)	9% (5/54)
RNA helicase A	0% (0/7)	24% (13/54)
Ro	14% (1/7)	41% (22/54)
La	0% (0/7)	11% (6/54)
Ku	0% (0/7)	7% (4/54)
Su	14% (1/7)	28% (15/54)
Any of the above	28% (2/7)	81% (44/54)^†^

*P = 0.0875, ^†^*P *= 0.0077 by Fisher's exact test.

## Discussion

SLE and CMV infection share common manifestations and a new infection or reactivation of CMV can mimic SLE [28]. CMV may also be considered responsible for flare or development of SLE in some cases [13,28-31]. Primary infection is characterized by positive IgM anti-CMV and negative IgG anti-CMV, followed by seroconversion to positive IgG anti-CMV; however, positive IgM anti-CMV is also seen frequently in patients with reactivation or reinfection of CMV [9,10,32]. CMV infection is a common complication in the immunocompromised host and in transplant patients under immunosuppressive therapy [10]. Whether reactivation of CMV is also common in SLE is controversial [25-27,33], because of a lack of standardized methods leading to inconsistency in the IgM anti-CMV assay and the PCR assay to detect CMV DNA.

In the present study, 95% of SLE patients were IgG anti-CMV(+). IgG anti-CMV detected at a high percentage (60% to 100%) in SLE patients was similar to the control population in most studies [11,25-27,34,35], although a few studies have reported a higher percentage of IgG anti-CMV in SLE patients versus control individuals [12,36].

The reported prevalence of IgM anti-CMV varies significantly from 5% to 45% [12,25-27,34], and was 33% in the present study. False positive results for IgM anti-CMV due to RF and other reasons have been reported [25-27]. In one study that reported IgM anti-CMV in 14% of SLE cases, 3/12 were considered an artifact after a RF neutralization assay [27]. Other studies interpreted IgM anti-CMV as false positive results based on negative PCR [25,26]. In contrast, one study detected CMV DNA by PCR in whole blood from 100% of SLE patients versus 72% in controls (*P *= 0.02) [12], suggesting a significant difference in sensitivity of the PCR assay. A recent study using immunostaining of CMV pp65 in peripheral blood leukocytes has suggested that reactivation of CMV is common among IgM anti-CMV(+) SLE patients under intensive immunosuppressive therapy [33]. The RF neutralization assay or PCR to detect CMV DNA was not performed in the present study; however, IgM anti-CMV was not associated with RF by laser nephelometry or ELISA (Figure 1), suggesting it was not directly due to RF. Furthermore, none of the IgM anti-CMV(+) group was positive for IgM antibodies to EBV–viral capsid antigen (data not shown). Also, the IgM anti-CMV(+) group did not have higher levels of IgM class autoantibodies to snRNPs, U1–70 k, dsDNA, chromatin, β_2_-glycoprotein I, or P peptide (Figure 1), indicating that IgM anti-CMV was not due to nonspecific IgM reactions in these patients. The majority of IgM anti-CMV therefore appears to be specific for the CMV assay and may be considered a reflection of reactivation or reinfection of CMV since all except one case had positive IgG anti-CMV.

Autoantibodies to snRNPs are found in ~40% of SLE patients, in 100% of mixed connective tissue disease and at lower prevalence in other systemic rheumatic diseases [37]. The autoimmune response to U1–70 k is considered characteristic of mixed connective tissue disease and an early autoimmune response in anti-U1RNP-positive patients [37,38]. CMV glycoprotein B/UL55 has a homology with the U1–70 k protein and can induce anti-U1–70 k antibodies in a mouse model [14,15]. Human vaccination of a live attenuated Towne vaccine, a recombinant CMV glycoprotein B vaccine or a glycoprotein B canarypox vectored vaccine (ALVAC-CMVgB) administered to CMV-seronegative subjects, however, did not induce antinuclear or anti-U1RNP antibodies [16,39]. Reports on the association between autoantibodies to the U1snRNPs and CMV infection in healthy subjects and SLE patients were inconsistent [11,16]. The study reporting a lack of association between anti-snRNP autoantibody production and CMV infection appears credible since they found no anti-U1RNP or anti-Sm positives among healthy individuals and only 2/80 were positive in the anti-U1–70 k ELISA [16]. The other study reported an increased prevalence of CMV infection among healthy individuals with anti-snRNP autoantibodies, however, with unusually high optical densities in the anti-U1RNP ELISA among healthy individuals (mean optical density 0.643 in the CMV(+) group versus 0.406 in the CMV(-) group) and a high prevalence of positive anti-U1RNP among healthy individuals (84% in the CMV(+) group and 24% in the CMV(-) group) [11], making the interpretation of this study difficult. Since we have only two CMV-seronegative SLE patients, our data cannot be compared with these studies.

In the present study, IgM CMV(+) patients had lower levels of anti-U1RNP/Sm and anti-U1–70 k autoantibodies versus anti-CMV IgM(-)IgG(+) patients (Figures 2 and 3). Anti-snRNPs and other lupus-related autoantibodies were more frequent in patients with high levels of IgG anti-CMV (Table 4). These observations are consistent with the role of immune response to CMV in lupus autoantibody production.

The mechanisms of the negative association of IgM anti-CMV and anti-snRNP autoantibodies are unclear. This does not appear to result from a difference in treatment between groups (Table 1); immunosuppressive treatment was not more common in patients of the IgM anti-CMV(+) group versus those of the IgM anti-CMV(-) group. Also, the reduced levels of antibodies in the former group were specific for IgG anti-U1snRNPs and anti-U1–70 k and were not observed in other specificities (Figures 1 and 2). The difference in IgG anti-snRNPs or U1–70 k units that reflect the amount of antibodies in the sera was 30-fold to 80-fold (Figure 2). All of these data suggest that the difference in levels of IgG anti-U1snRNPs and U1–70 k antibodies between groups cannot be a simple reflection of different total IgG levels. It is tempting to speculate that reactivated/reinfected CMV or its products play a role in downregulating anti-snRNP autoantibodies. This may sound strange since most previous studies were focused on the role of viruses in inducing or enhancing autoantibodies in SLE [3]; however, viruses have various mechanisms to inhibit host immune function in order to survive and escape from elimination by the host immune system. In fact, viral anti-inflammatory and immune-modulating proteins have been applied to treat inflammatory and immune disorders [40]. There are several possible mechanisms that may explain inhibition of anti-snRNP autoantibody production by CMV.

First of all, U1RNA can stimulate type I interferon (I-IFN) production via TLR7 [41] and anti-snRNP autoantibody production is TLR7 and I-IFN dependent [42,43]. The antagonistic effects of TLR7 and TLR9 stimulation reported recently [44,45] are of particular interest in speculating that the binding of CMV DNA to TLR9 may interfere in the TLR7-dependent anti-snRNP autoantibody production.

A second consideration is that CMV has various mechanisms to escape from the attack by a host immune system [46-48], including suppression of I-IFN production [49]. CMV IL-10 or host IL-10 induced by reactivated or reinfected CMV may shift the cytokine balance towards T helper type 2 and show antagonistic effects on IFNγ-dependent production of anti-snRNP autoantibodies [50].

Preferential effects on anti-snRNP autoantibody production are another interesting point that cannot be explained by available information. This situation may not be so unusual, however, considering that the same environmental or endogenous factor can exhibit different effects on different specificities of lupus autoantibodies [51-54]. Any immunological effect of CMV or a combination of the immunological effects of CMV [49] can shift an environment to one unfavorable for production of anti-snRNPs, leading to reduced levels of anti-snRNP autoantibodies in SLE patients with reactivated CMV.

On the other hand, a strong immune response against CMV proteins may induce or enhance anti-snRNP autoimmune response via molecular mimicry, as shown in mouse models [14,15]. Depending on the balance of immune response to CMV versus the immunosuppressive effects of CMV, therefore, *in vivo *biological effects of CMV may work towards enhancing or suppressing immune and autoimmune responses. Positive association of high levels of IgG anti-CMV and IgG lupus autoantibodies and negative association of IgM anti-CMV (reflecting reactivation of CMV) and IgG anti-snRNPs antibodies may be consistent with this possibility.

Coexistence of anti-Su with anti-snRNPs or anti-Ro was also an interesting finding. Like the U1RNA component of snRNPs, the Y RNAs of Ro antigens induce I-IFN via TLR7 stimulation [41]. Anti-snRNPs and anti-Ro are both associated with production of high levels of I-IFN in SLE [55]. The Su antigen was recently identified as Ago2, a component of RNA interference machinery enriched in GW/P bodies containing macromolecular complex with mRNA and microRNA [56]. Anti-Su production in an animal model is also TLR7 dependent, similar to anti-snRNP production [43]. Three autoantibodies that appear to coexist often in the anti-CMV IgM(-)IgG(+) subset of SLE cases (Table 3) are therefore all involved in TLR7 stimulation and/or I-IFN production, suggesting common mechanisms in production.

## Conclusion

Our findings suggest the potential role of CMV in regulation of autoantibodies to snRNPs and may provide a unique insight to understanding the mechanisms of production of lupus autoantibodies. Longitudinal studies carefully monitoring the kinetics and serology of CMV and autoantibodies will be necessary to understand the role of CMV in lupus autoantibody production.

## Abbreviations

BSA: bovine serum albumin; CMV: cytomegalovirus; dsDNA: double-stranded DNA; EBNA: EBV nuclear antigen; EBV: Epstein–Barr virus; ELISA: enzyme-linked immunosorbent assay; IFN: interferon; I-IFN: type I interferon; IL: interleukin; mAb: monoclonal antibody; PCR: polymerase chain reaction; RF: rheumatoid factor; SLE: systemic lupus erythematosus; snRNP: small nuclear ribonucleoprotein; TLR: Toll-like receptor; U1–70 k: U1snRNP-70 kDa protein; VCA: viral capsid antigen.

## Competing interests

The authors declare that they have no competing interests.

## Authors' contributions

CAPS and MS helped to design the study, perform experiments, and write the manuscript. EKLC designed and expressed recombinant proteins and helped write the manuscript. WCC expressed and purified recombinant proteins. JFMV, GOB, EOR, RENH, MSP and ACC helped to design and perform experiments. MVDM designed the study, performed experiments, and wrote the manuscript. All authors read and approved the final manuscript.
